# Nine quick tips for pathway enrichment analysis

**DOI:** 10.1371/journal.pcbi.1010348

**Published:** 2022-08-11

**Authors:** Davide Chicco, Giuseppe Agapito

**Affiliations:** 1 Institute of Health Policy Management and Evaluation, University of Toronto, Toronto, Ontario, Canada; 2 Dipartimento di Giurisprudenza Economia e Sociologia, Università Magna Graecia di Catanzaro, Catanzaro, Italy; McGill University, CANADA

## Abstract

Pathway enrichment analysis (PEA) is a computational biology method that identifies biological functions that are overrepresented in a group of genes more than would be expected by chance and ranks these functions by relevance. The relative abundance of genes pertinent to specific pathways is measured through statistical methods, and associated functional pathways are retrieved from online bioinformatics databases. In the last decade, along with the spread of the internet, higher availability of computational resources made PEA software tools easy to access and to use for bioinformatics practitioners worldwide. Although it became easier to use these tools, it also became easier to make mistakes that could generate inflated or misleading results, especially for beginners and inexperienced computational biologists. With this article, we propose nine quick tips to avoid common mistakes and to out a complete, sound, thorough PEA, which can produce relevant and robust results. We describe our nine guidelines in a simple way, so that they can be understood and used by anyone, including students and beginners. Some tips explain what to do before starting a PEA, others are suggestions of how to correctly generate meaningful results, and some final guidelines indicate some useful steps to properly interpret PEA results. Our nine tips can help users perform better pathway enrichment analyses and eventually contribute to a better understanding of current biology.

## Introduction

Pathway enrichment analysis (PEA), also known as functional enrichment analysis or overrepresentation analysis, is a bioinformatics procedure that identifies specific biological pathways as being particularly abundant in a list of genes [1].

Biological pathways describe molecular activities or roles of genes of different kinds. Pathway databases can be specific (HumanCyc [2] for metabolic pathways and LIPEA for lipid functions [3], for example) or more general purpose (KEGG [4], Reactome [5], and WikiPathways [6], for example). Molecular functions can also be represented in a structured hierarchy: The Gene Ontology (GO) [7], for example, contains structured biomolecular annotations that indicate biological processes, molecular functions, or cellular components, connected in directed acyclic graphs.

Several statistical methods can be used to associate the most enriched biological pathways in the input gene list and take into account the number of genes and the likelihood of a pathway to be found enriched. g:Profiler g:GOSt [8–11], for example, uses a modified Fisher’s exact test [12–15] to estimate abundance of the genes considering the frequency of the genes in the pathways’ database. g:Profiler g:GOSt then proposes three different methods for computing multiple testing correction for *p*-values (g:SCS, Bonferroni correction, or Benjamini–Hochberg false discovery rate (FDR); S2 Text) [10,16].

Multiple PEA tools are available in the scientific literature, both as web tools and as stand-alone software programs. Some of them employ multiple databases, while others use only one, but they all have the same goal: take an input gene list and associate biological pathways with the larger gene overlap than the one obtained by chance.

Even if a PEA can be done easily, it is also easy to make mistakes that can generate overoptimistic or misleading results. We therefore propose these nine quick tips that can help beginners and inexperienced users perform a PEA properly, by avoiding common errors or pitfalls.

Other authors reported potential problems of functional enrichment analysis [17–21] and described best practices in the past [22, 23], but we believe that our guidelines are easier to follow and to understand by all users, including students and beginners.

### Tip 1: Before starting, clarify which analysis you would like to perform

As simple as it might sound, the first step for a sound and robust PEA is about making up your mind: What analysis do you plan to perform? The answer to this question depends mainly on the type of scientific problem you would like to solve and on the type of data you have.

#### What analysis type

Several different enrichment analyses are available in the bioinformatics landscape; even if most of them have significant differences, sometimes their names are used as synonyms, increasing confusion in the scientific literature. PEA, which is the main topic of this article, is sometimes called functional enrichment analysis. These two names indicate the same procedure: the identification of enriched biological pathways (also called “biological functions”) in a list of biomolecular entities (usually genes, but also microRNAs or metabolites), through statistical methods.

PEA methods can also be classified into overrepresentation analysis (ORA) and gene set enrichment analysis (GSEA) approaches. The ORA name highlights the importance of the biological functions that are overrepresented in a group of genes with respect to their role in the human genome [24]. GSEA is both the name of a bioinformatics tool developed and released by scientists at University of California San Diego (UCSD) and Broad Institute [25–27] and the name of the type of analysis they invented. The authors of Enrichr [28–30], for example, define its goal as GSEA.

Some users refer to GSEA and PEA as synonyms [31,32]. Each ORA and GSE approach can be categorized into the competitive and/or self-contained classes based on the null hypothesis. Competitive methods compute *p*-values assuming the genes independence hypothesis is not always true, whereas self-contained methods assume that genes in the gene list are equally associated with phenotype as genes not listed, yielding many relevant genes (like ROAST [33], for example). GSEA approaches are considered a mix of self-contained and competitive methods, since they permute only the genes’ class labels (for example, phenotypes) into the pathway, or permute all the genes’ class labels for each pathway, comparing the pathway gene set with the query gene set, depending on the parameters chosen [34].

Thus, GSEA methods can perform both self-contained and competitive hypothesis tests by altering how permutation is done for testing the null hypothesis. It is worthy to note that many PEA tools provide both options, ORA and GSEA. ORA methods differ from GSEA because they only consider the query gene set of interest and need a strict cutoff to classify genes as up- and down-regulated; thus, it is advisable to choose GSEA methods when there is uncertainty about the cutoff value. BioPAX-Parser (BiP) [35], pathDIP [36,37], SPIA [38], CePaORA [39], and PathNet [40] are competitive methods, whereas CePa [39] and GSEA [25–27] are self-contained methods. More precisely, the main difference between the GSEA approaches and the ORA approaches is the output: GSEA indicates the pathways that are enriched in genes located at both extreme ends of a ranked gene list, and a higher ranked pathway indicates that more genes are located at the very top or at the very end of this list.

Conversely, ORA outputs all pathways enriched in the query gene list as a whole, and mainly uses a nonranked list (except one option in g:Profiler g:GOst using a minimum hypergeometric value-based method). Therefore, the focus of ORA methods is the gene set, while the focus of GSEA techniques is the ranked pathways list. In this article, we will consider this distinction even if, as we mentioned earlier, the terms GSEA or PEA are often used as synonyms in the scientific literature. Furthermore, topology-based PEA (TPEA) is an advanced PEA that takes into account the hierarchical topology of the analyzed genes, such as the interactions between genes and gene products [24,41–43].

Even if these methods generally produce more precise results, they suffer from the limitation of using a gene topology based on the single cell type in use [24]. Moreover, the topologies of the genes are far from being final and might change as the general biology understanding advances. Lastly, researchers refer to chromosome region enrichment analysis or genomic enrichment analysis to PEA tools that read lists of genomic regions as input, rather than lists of genes. These analyses first associate genes to genomic regions and then retrieve their corresponding biological pathways. GREAT [44], BEHST [45], and Poly-Enrich [46] belong to this category. We report the complete list of PEA tools mentioned in this article in S1 Table. Unlike what some unexperienced PEA users think, it is important to note that PEA does not give clues about the active or inhibited status of the pathways. More appropriately, PEA provides information about how genes help carry out pathways.

#### Which data type

As it is easy to understand, the type of analysis depends also on the type of the data one would like to analyze. For unordered lists of genes, researchers can use g:Profiler g:GOSt [8–10], Enrichr [28,29], and BioPAX-Parser [35,47]. If the genes are ranked, g:Profiler g:GOSt can treasure this information and generate rank-based functional enrichment results. If the input data are gene expression levels, they can be analyzed through GSEA [27]. pathDIP [37], instead, can assist with curated analyses based on scientific literature.

If one would like to have topological scores to rank cross-enriched pathways using more pathway databases, cPEA [48] might be the best tool choice. GeneTrail [49–52] can be useful for results related to epigenetics, while NoRCE [53] serves well for investigating noncoding RNAs.

Another aspect to keep in mind is the format of the data one would like to analyze, and their specific representation. Different models can represent multiple biochemical reactions responsible for biological functions and pathways. Usually, signaling or metabolic pathways are considered sets of genes interacting in a coordinated way to accomplish a given biological function or process. For instance, in a standard signaling pathway, KEGG [4] uses nodes to represent genes or gene products and edges to define signals, such as activation or inhibition, going from one gene to another. A common metabolic pathway would be depicted with nodes to represent biochemical compounds and edges to represent reactions that transform one or more compounds into other compounds. Enzymes coded by genes usually accomplish these reactions. Therefore, genes or their products are associated with edges rather than nodes in a metabolic pathway, like a signaling pathway. Keep in mind that the immediate impact of this difference is that many techniques cannot be applied directly to all available pathway types (S3 Text).

To summarize, before running any analysis, spend some time studying which scientific question you would like to answer, which data you have for your study, and of which data type they consist. The answers to these questions will help you determine the most suitable enrichment analysis to use.

### Tip 2: Ensure the quality of your input genes or genomic regions

The popular saying “Garbage in, garbage out” summarizes a key pillar of computer science: If the quality of data inputed into a computational system or method is poor, the output results will also be of bad quality [54,55]. No matter how efficient and robust a computational method is, to have meaningful results at the end of a computational analysis, data must be of good quality at input. This rule is valid for all computer science, including bioinformatics, and is true for PEA as well.

Before starting a functional analysis, double-check the input list of genes or genomic regions: study how that list was generated, with which tools and when. What criteria were employed in selecting those genes or chromosome regions? Was a scientific article related to that list published recently? If yes, it is a good idea to read it carefully. In a nutshell, ensure that the gene list or the genomic region list that you plan to use for your PEA was assembled in a meaningful, thorough, precise way, with a valid scientific rationale.

If you notice that the input list was generated in an obscure, odd, illogical way, discard it and focus your attention on another gene list. Let us suppose you would like to investigate a diagnostic genetic signature for breast cancer, derived from microarray gene expression. You read the article related to this signature and notice that the authors used 3 datasets generated on three different microarray platforms (Affymetrix, Illumina, and Agilent, for example), without doing any batch effect correction [56,57]. It is clear that this study contains a preprocessing mistake and its results should be discarded or at least treated with caution. In cases like this, we suggest investigating further this list of genes or even avoiding any functional enrichment analysis and look for another genetic signature.

Another red flag for a proposed gene list would be the absence of a validation on an external data cohort. If a gene list was proposed in a study involving only one dataset, it is probably not reliable enough for prognostic or diagnostic scopes.

If some gene symbols are not recognized by the PEA tool, we suggest to look for their Entrez Gene ID’s through g:Profiler g:Convert [9] or to look for their symbol aliases on Gene Cards [58–60].

Do not run a functional enrichment analysis on any gene list “just to see if anything comes out”: If “anything” comes out, it is probably misleading. Only when you are sure about the quality of your gene list you can proceed and start your PEA.

Even if the gene list is well curated, some technical issues can still occur, for example, with the gene symbols.

### Tip 3: Use multiple PEA tools, not only one

What we see in multiple studies involving phases of PEA is the habit of employing a single PEA tool. Many bioinformatics and biomedical researchers, in fact, learn how to use one PEA tool well and then stick with it forever by including an analysis done with it in most of their published studies. This approach has several limitations, because using a unique PEA tool of course generates results that are relegated to the databases associated with that specific PEA software.

Even if it seems obvious, we suggest all the practitioners performing a functional enrichment analysis phase to employ at least two different PEA tools. Having results coming from different sources and methods is pivotal: Some results will be confirmatory, some will be complementary, and some might even be discordant. Seeing two sides of a PEA analysis surely can give a user the possibility to learn more about the pathways associated with the input genes.

For example, if the user had an unranked list of gene symbols, we would suggest to apply g:Profiler g:GOSt [10], Enrichr [28], and GeneTrail [52] to it, and then compare their results. Each of these three PEA tools share common databases (the Gene Ontology, for example) but also have specific ones. A user could then analyze their results first to verify if the common pathways are found by all the three methods, and then to analyze the unique terms found. The comparison between the output pathways generated by different PEA tools can be tricky but can reveal essential information about the analyzed gene list.

A quick, straightforward way to compare the enrichment results is to verify if they contain pathways with the same name. This solution could be insufficient since pathways belonging to various databases may have distinct names and might be structured in a redundant, partially overlapping manner in some other databases. This aspect is a well-known complication in PEA due to the lack of a unique standard to represent and store biological pathway data. Consequently, many available software tools can only deal with a single pathway database. To perform pathway enrichment by employing more than a single database, users can employ cPEA [48], a software tool able to deal with several pathway databases using the BioPAX language [61] to store and represent pathways. Or they can use BiP [47] by selecting the “Whole PathwayCommon Data” option that will perform cross enrichment using the whole collection of automatically downloaded locally Pathway Common databases [62] coded in BioPAX. It is worth noticing that evaluating the similarity among pathways may be helpful to compare the genes within each pathway.

Moreover, we describe two possible ways to compare, consolidate, and validate pathways in S1 Text.

### Tip 4: Document all your PEA tests and their details

For each PEA software used, keep track of its version, of its parameters’ arguments, and of all its details [63,64]. Write this precious information in a notebook [65], and then include it in the supplementary information of the article about the given PEA study. This step is important for you and your future analysis comparison, but also for the reproducibility of your research study [66,67].

In Box 1, we report an example of details and information regarding a functional enrichment analysis made with g:Profiler g:GOSt that should be manually written by a bionformatician in her or his notes. The user should save these pieces of information in addition to saving the full results of the PEA tests, of course. In particular, we recommend to take note of the version and last update of the databases employed: Since they change quite often, some biological annotations can become obsolete soon, with negative consequences on the scientific outcomes [68].

**Box 1: Example of PEA test details**

My test ID: 2022-02-04, h10:02 EST.

My input genes: AK4, ALDOC, EGLN1, FAM162A, MTFP1, PDK1, PGK1.

My input genes’ type: gene symbols.

Source: D. Cangelosi and colleagues [69].

Disease: neuroblastoma.

Tool: g:Profiler g:GOSt.

Access: online via Google Chrome browser.

Version: e104_eg51_p15_3922dba.

URL: https://biit.cs.ut.ee/gprofiler/gost

Organism: *Homo sapiens*.

Query: unordered genes.

Statistical domain scope: only annotated genes.

Significance threshold: g:SCS threshold.

User threshold: 0.005.

Data sources: default.

All the other parameters: default.

My output file(s) name(s): gprofiler_gost_NB_2022-02-04_h1002_output.csv

My output file(s) folder: /home/davide/PEA_analyses/neuroblastoma/

My output file(s) location: bioinformatics-laptop-2021 (Dell Latitude E5420).

The last part of Box 1 regarding the output file name and location should not be included in the manuscript, of course, but should be written in the user’s notebook. This piece of information will be invaluable in the future.

### Tip 5: Always use the corrected *p*-value, and not the nominal one

As we explained earlier, pathway enrichment analyses include statistical steps that rank the output pathways by abundance in the gene list and express their enrichment through a probability value called *p*-value. The closer to zero this *p*-value is, the more significant the result is. However, since we know that genes are unevenly annotated in the biological databases, using the simple nominal *p*-values would easily generate misleading results. Two different Gene Ontology annotations, for example, might end up having *p* = 0.001 in the PEA results, and the user might think they are enriched in this gene list. However, these *p*-values might just be related to the fact that one of the two is actually enriched, while the other is just annotated to few genes in the Gene Ontology database.

Additionally, since one *p*-value needs to be calculated for each term, it is very likely that some terms might end up having a significant *p*-value just by chance.

To alleviate this issue, we recommend using the adjusted *p*-value for multiple testing, sometimes also called corrected *p*-value and indicated as *p.adj* [70].

In the hypothesis testing, we have our hypothesis that says that some variables are correlated, and a null hypothesis that states there is no relationship between them [71]. If our test’s *p*-value is significant, we can reject the null hypothesis and claim that our hypothesis is true. The issue is that, with many variables and, therefore, multiple hypotheses to test, there is a higher chance to make at least one type 1 error that is to reject the null hypothesis even if the null hypothesis is actually true. This result would be misleading and can be alleviated through methods for multiple testing error correction or adjustment.

This adjustment limits the family error rate or the FDR and therefore improves the quality of the PEA outcomes. An example of technique for the family error rate correction is the Bonferroni method [72], while a common procedure for FDR correction is the Benjamini–Hochberg procedure [73]. The terms adjusted *p-values*, *corrected p-values*, and *false discovery rate (FDR)* values are often used as synonyms in the scientific literature.

Additionally, following a recent debate on the best practices for computational statistics [74], we suggest using the adjusted *p*-value threshold at 0.005 (that corresponds to 5 × 10^−3^), as recommended by Benjamin and colleagues [75].

We know that the significance of the results cannot be indicated by a single threshold for all possible PEA experiments: The significance of the pathways, instead, depends on the input data, on the size of the gene list, on the tool and method employed, on the databases used, and on the nonindependence between the genes. We therefore suggest using the *p*.*adj* < 0.005 threshold for a first strict analysis of the results, and then repeating the test by using a more permissive threshold such as *p*.*adj* < 0.01, and then again with an even higher threshold, such as the traditional *p*.*adj* < 0.05. Based on the characteristics of the experiment, results found by one particular threshold might be more suitable than results found with other thresholds.

In any case, the results found in this phase should be then validated through wet lab experiments or a literature review (Tip 8) and reviewed by a wet lab biologist (Tip 9), since these steps would avoid publication of many false findings [76,77].

We believe this tip is true not only for bioinformatics, but also for all the scientific studies involving statistics and probability values.

### Tip 6: Keep in mind that your PEA results can be strongly affected by the statistical tests and the visualization techniques you use

#### Statistical tests

As we mentioned earlier, each PEA software tool uses a different statistical method to identify the biological pathways enriched in a set of input genes. These statistical techniques associate a corrected probability value (*p*-value) to each pathway, which indicates its importance: the lower the adjusted *p*-value is, the more the pathway is enriched in genes from the query gene list compared to all genes.

Pathways are not equal in their number of genes they contain, and some contain a limited number of curated genes, but therefore can be very relevant in a PEA analysis if found enriched in a high percentage of input genes.

Different statistical techniques, however, can generate different results, and this is something users should always keep in mind. We describe an example of different results obtained on g:Profiler g:GOSt when using different statistical tests in S2 Text, and we report the list of the statistical methods of the PEA tools mentioned in this article in S1 Table.

Our general advice for this task is to keep in mind that different statistical methods can generate different results, so avoid blind use of any statistical test. Study which statistical method can be more suitable for your analysis and why, and then apply it.

#### Visualization

Scientific visualization is a key pillar of bioinformatics and of modern scientific research [78]. Proper visualization plots do not only represent the data or the results observed in an experiment, but they can also provide alternative, new insights about the data themselves [79].

The visualization step of a PEA, although fundamental, is sometimes underrated by inexperienced users. On the contrary, we believe this phase is vital for the interpretation of the PEA results. Following what we suggested in Tip 4, we advise all PEA practitioners to employ multiple tools for this task. Moreover, the key point to keep in mind during this phase is that different visualization tools and styles can highlight different scientific aspects of the results and therefore unveil unexpected biological novelty that would have been unnoticed otherwise.

Visualization of PEA results can be useful and advantageous because it easily allows users to quickly detect the main enriched functional subjects, which they can then use to interpret the enrichment results. This identification of the functional subjects and their interpretation would be more difficult without a visualization step. Moreover, several useful PEA visualization techniques allow users to deal with redundancy of enrichment results by grouping together similar processes and pathways into common functional themes.

Enrichment Maps [80] and enrichplot [81] for biological pathways, AutoAnnotate [82] for networks, and REVIGO [83] and CirGO [84] for GO annotations are few examples of different visualization techniques and contents. Network visualization techniques can also be used to detect a lower adjusted *p*-value threshold (Tip 5).

To recap, avoid blind use of visualization techniques: understand the available ones and choose the most suitable one for your case.

### Tip 7: Consider using subgroups of correlated genes instead of all your input genes

A common practice in PEA is to take all the genes derived from an experiment or a previous analysis and to use them all as input in a PEA tool. This is surely a good thing to do when the users do not know any hierarchy or relationship between the input genes, but it can also produce many gene–pathway associations that might turn out to be irrelevant or even misleading in the end. Additionally, using many input genes could produce a large number of general pathways in the results, such as “signaling pathway” available in KEGG [4] and Reactome [5], for example, which do not improve our understanding of the affected biological processes and functions. Some PEA tools give the possibility to exclude these generic terms from the results, but not all of them.

Instead of using all genes as input for the PEA, we therefore suggest bioinformatics practitioners to detect subgroups of correlated genes and perform the PEA on each of these subgroups alone.

Subgroups of correlated genes can be found, for example, through protein–protein interaction networks’ tools such as IID [85–88], STRING [89–92], GeneMANIA [93–97], or Reactome Functional Interaction Network (Reactome FI) [98,99]. These software programs are able to cluster together groups of genes that might share a common physical interaction in their databases. Woodwarda and colleagues [100] recently released a GSEA tool enhanced with epistatic interactions [101], which might be of interest for this scope.

To this end, some R packages have been recently released: pathfindR [102] and netGO [103], which exploit the protein–protein interaction networks to produce more accurate PEA results.

Using these groups of genes, which are already correlated between each other by sharing the same physical interactions, would probably detect more precise biological pathways as output of the PEA.

### Tip 8: Use the (recent) scientific literature to review your PEA results

The results you obtained with the PEA tools used are surely interesting and useful, but they are likely based on databases and datasets collected some months or even years ago. Therefore, your results might not be as novel as the recent scientific literature: Some new studies about pathway–gene associations might have been published between the release of the databases employed by the PEA tools used and when your PEA was executed.

To verify your findings, we therefore suggest any practitioner to manually perform a literature search and look for scientific studies published about the significant genes–pathways associations found by the functional enrichment analysis and about the role of the genes inside the enriched pathways found.

The search can be done by using the pathways and the genes as keywords on Google Scholar [104] and PubMed [105]. We also suggest to search on the preprint servers such as bioRxiv [106] and arXiv Quantitative Biology [107], although the fact that these preprint documents are not peer reviewed should be kept in mind. This phase can help alleviate the problem of outdated gene annotations [68].

However, we know that sometimes the list of genes and the list of pathways are so large that manually looking for at least one article about each of them would take too much time. As a rule of thumb, we therefore suggest the users to investigate at least the top twenty genes–pathways associations in the literature. Alternatively, the user could filter the genes by known importance: They could study their input list of genes, identify some frequently seen genes that they already saw in the literature, and investigate their pathways found by the PEA. In any case, we invite users to verify PEA results by looking at the role of the genes shared in the top PEA output pathways to precisely define the biological functions targeted by the gene list (S1 Text).

### Tip 9: Ask a wet lab biologist or a clinician to review your PEA results

After looking for evidence about the results of a PEA with scientific literature (Tip 8), we believe one additional step is needed: To further validate the PEA results achieved, a wet lab biologist or a clinician should review these results and clearly say if they make sense or if they contain mistakes or inappropriate information.

Similarly to what is suggested for machine learning studies [108], we therefore suggest that all computational biologists, after performing the PEA, contact a biology researcher and ask for a review of their PEA results. This person should not be a user or a computational biologist but should have a degree in traditional biology or medicine and should be familiar with scientific results obtained in the wet lab.

The point of view of this expert will surely provide interesting considerations and feedback regarding the PEA results and will highlight some aspects that maybe the user might have overlooked. If possible, one can also consider asking this person to perform some wet lab validation of the results found through the PEA. We intended this list of quick tips for computational analyses, but it goes without saying that a precise biological validation made in a wet lab would be extremely useful and even more relevant than any literature review.

## Conclusions

PEA is a pivotal step of bioinformatics studies that highlights the most relevant biological functions associated with gene lists. In the last decade, huge computational resources and numerous web tools available online made this analysis type easy for anyone to perform. However, it also became easy to make mistakes by using PEA tools, data, or results that might not address the original scientific scope properly. Following the recent debates emerged in machine learning and biomedical informatics communities [108–116], we propose these nine quick tips that can be used as a checklist for any computational user running a functional enrichment analysis.

## Supporting information

S1 Table List of the PEA tools mentioned in this study. (PDF)

S1 Text Description of the validation of genes inside the pathways. (PDF)

S2 Text Example of usage of different statistical tests in g: Profiler g:GOSt. (PDF)

S3 Text Description of pathway data conversion. (PDF)
