# Supplementary material for: Nine quick tips for pathway enrichment analysis
Source: PLoS Comput Biol. 2022 Aug 11;18(8):e1010348. doi: 10.1371/journal.pcbi.1010348 (PMC9371296; doi:10.1371/journal.pcbi.1010348)
Supplement: S1 Table — (PDF) [file pcbi.1010348.s001.pdf]

S1.1 List of PEA tools

tool	input data types	statistical methods	reference
BEHST	ordered chromosome regions	modified Fisher's exact test	[1]
BioPAX-Parser	unranked gene list	hypergeometric test	[2]
CePa	pathway nodes	gene-level statistics	[3]
Enrichr	unranked gene list	Fisher's exact test variant	[4, 5]
g:Profiler g:GOSt	ranked or unranked gene list	modified Fisher's exact test	[6–9]
GeneTrail	unranked gene list	dynamic programming algorithm	[10–13]
GREAT	ordered chromosome regions	binomial and hypergeometric tests	[14]
GSEA	gene expression or ranked gene list	empirical phenotype-based permutation test	[15]
NoRCE	ordered chromosome regions and unranked gene list	hypergeometric test, Fisher exact test, binomial test, χ^2 test	[16]
pathDIP	unranked gene list	Fisher's exact test followed by correction	[17, 18]
PathNet	gene expression	hypergeometric test	[19]
Poly-Enrich	ordered chromosome regions	hybrid test	[20]
SPIA	$\log_2$ fold-changes of the differentially expressed genes	moderated t-test	[21]

Table S1. List of the PEA tools mentioned in this study. For each tool we report its name linked to its URL, input data types, statistical methods to calculate the significance of the enrichment , and reference. Ranked: derived from a ranking. Ordered: sorted on the genomic position.

References

1. Chicco D, Bi HS, Reimand J, Hoffman MM. BEHST: genomic set enrichment analysis enhanced through integration of chromatin long-range interactions. bioRxiv. 2019;168427:1–29.

2. Agapito G, Pastrello C, Guzzi PH, Jurisica I, Cannataro M. BioPAX-Parser: parsing and enrichment analysis of BioPAX pathways. Bioinformatics. 2020;36(15):4377–4378.

3. Gu Z, Wang J. CePa: an R package for finding significant pathways weighted by multiple network centralities. Bioinformatics. 2013;29(5):658–660.

4. Chen EY, Tan CM, Kou Y, Duan Q, Wang Z, Meirelles GV, et al. Enrichr: interactive and collaborative HTML5 gene list enrichment analysis tool. BMC Bioinformatics. 2013;14(1):1–14.

5. Kuleshov MV, Jones MR, Rouillard AD, Fernandez NF, Duan Q, Wang Z, et al. Enrichr: a comprehensive gene set enrichment analysis web server 2016 update. Nucleic Acids Research. 2016;44(W1):W90–W97.

6. Reimand J, Kull M, Peterson H, Hansen J, Vilo J. g:Profiler—a web-based toolset for functional profiling of gene lists from large-scale experiments. Nucleic Acids Research. 2007;35(suppl_2):W193–W200.

7. Reimand J, Arak T, Vilo J. g:Profiler—a web server for functional interpretation of gene lists (2011 update). Nucleic Acids Research. 2011;39(suppl_2):W307–W315.

8. Reimand J, Arak T, Adler P, Kolberg L, Reisberg S, Peterson H, et al. g:Profiler—a web server for functional interpretation of gene lists (2016 update). Nucleic Acids Research. 2016;44(W1):W83–W89.

9. Kolberg L, Raudvere U, Kuzmin I, Vilo J, Peterson H. gprofiler2–an R package for gene list functional enrichment analysis and namespace conversion toolset g:Profiler. F1000Research. 2020;9.

10. Keller A, Backes C, Al-Awadhi M, Gerasch A, Küntzer J, Kohlbacher O, et al. GeneTrailExpress: a web-based pipeline for the statistical evaluation of microarray experiments. BMC Bioinformatics. 2008;9(1):1–6.

11. Backes C, Keller A, Kuentzer J, Kneissl B, Comtesse N, Elnakady YA, et al. GeneTrail—advanced gene set enrichment analysis. Nucleic Acids Research. 2007;35(suppl_2):W186–W192.

12. Stöckel D, Kehl T, Trampert P, Schneider L, Backes C, Ludwig N, et al. Multi-omics enrichment analysis using the GeneTrail2 web service. Bioinformatics. 2016;32(10):1502–1508.

13. Gerstner N, Kehl T, Lenhof K, Müller A, Mayer C, Eckhart L, et al. GeneTrail 3: advanced high-throughput enrichment analysis. Nucleic Acids Research. 2020;48(W1):W515–W520.

14. McLean CY, Bristor D, Hiller M, Clarke SL, Schaar BT, Lowe CB, et al. GREAT improves functional interpretation of *cis*-regulatory regions. Nature Biotechnology. 2010;28(5):495–501.

15. Subramanian A, Kuehn H, Gould J, Tamayo P, Mesirov JP. GSEA-P: a desktop application for Gene Set Enrichment Analysis. Bioinformatics. 2007;23(23):3251–3253.

16. Olgun G, Nabi A, Tastan O. NoRCE: non-coding RNA sets cis enrichment tool. BMC Bioinformatics. 2021;22(1):1–17.

17. Rahmati S, Abovsky M, Pastrello C, Jurisica I. pathDIP: an annotated resource for known and predicted human gene-pathway associations and pathway enrichment analysis. Nucleic Acids Research. 2017;45(D1):D419–D426.

18. Rahmati S, Abovsky M, Pastrello C, Kotlyar M, Lu R, Cumbaa CA, et al. pathDIP 4: an extended pathway annotations and enrichment analysis resource for human, model organisms and domesticated species. Nucleic Acids Research. 2020;48(D1):D479–D488.

19. Dutta B, Wallqvist A, Reifman J. PathNet: a tool for pathway analysis using topological information. Source Code for Biology and Medicine. 2012;7(1):1–12.

20. Lee CT, Cavalcante RG, Lee C, Qin T, Patil S, Wang S, et al. Poly-Enrich: count-based methods for gene set enrichment testing with genomic regions. NAR Genomics and Bioinformatics. 2020;2(1):lqaa006.

21. Tarca AL, Draghici S, Khatri P, Hassan SS, Mittal P, Kim Js, et al. A novel signaling pathway impact analysis. Bioinformatics. 2009;25(1):75–82.
