# Supplementary material for: Nine quick tips for pathway enrichment analysis
Source: PLoS Comput Biol. 2022 Aug 11;18(8):e1010348. doi: 10.1371/journal.pcbi.1010348 (PMC9371296; doi:10.1371/journal.pcbi.1010348)
Supplement: S1 Text — (PDF) [file pcbi.1010348.s002.pdf]

S1.2 Validation of the genes inside the pathways

Even when the enrichment results are plausible, they might fail to reveal all the biological nuances. It is consequential to notice that enrichment focuses on genes showing considerable expression level differences between the conditions, while the genes with modest changes may go unnoticed. For instance, even small changes in expression levels in a signaling pathway may have profound biological consequences.

Hence, enrichment results should be further validated and consolidated by exploring two different paths. One path uses the available literature to search for evidence that can elucidate the role of the enriched pathways in the condition under investigation. This path is suitable for novel PEA users like students or biologists without computational skills.

The available literature can be explored manually by using public knowledge repositories like PubMed to search for evidence that can contribute to confirming the effectiveness of enrichment results, or even with the help of additional software such as PubTor, that can contribute to simplifying and speeding up the search for pieces of evidence, making a tedious and long manual searching process easier. Indeed, PubTator Central (PTC) is a Web-based system [1–3] providing automatic annotations of biomedical concepts such as genes, pathways, and mutations in PubMed abstracts and PMC full-text articles. PTC is free and can be accessed interactively through a web browser, programmatically via RESTful API, or downloaded in bulk via FTP.

At the same time, the second path uses statistical methodologies to reveal hidden biological functions nuances, a path recommended especially to skilled users. Users must implement an automatic or manual analysis workflow to handle the enriched results. The workflow's first step concerns identifying the pathway's input genes contributing to the enrichment. Next, the figured out genes can be assessed manually or through scripts in Gene Ontology (GO) [4–8] to get more knowledge about their possible functional roles. Moreover, PTC can provide further annotations about the relevant genes and their involvement in pathways.

Additionally, it is worth mentioning that Geistlinger and colleagues [9] have recently released *GSEABenchmarkeR*, an R package for benchmarking gene set enrichment analysis results.

References

1. US National Library of Medicine (NLM). PubTator - Discover biomedical entities in more than 30 million biomedical publications; 2022. `https://www.ncbi.nlm.nih.gov/research/pubtator/` URL visited on 21st April 2022.

2. Wei CH, Kao HY, Lu Z. PubTator: a web-based text mining tool for assisting biocuration. Nucleic Acids Research. 2013;41(W1):W518–W522.

3. Wei CH, Allot A, Leaman R, Lu Z. PubTator Central: automated concept annotation for biomedical full text articles. Nucleic Acids Research. 2019;47(W1):W587–W593.

4. The Gene Ontology Consortium. The Gene Ontology resource: 20 years and still GOing strong. Nucleic Acids Research. 2019;47(D1):D330–D338.

5. The Gene Ontology Consortium. The Gene Ontology Resource; 2022. `http://geneontology.org/` URL visited on 21st April 2022.

6. Blake JA. Ten quick tips for using the Gene Ontology. PLOS Computational Biology. 2013;9(11):e1003343.

7. Chicco D, Masseroli M. Software suite for gene and protein annotation prediction and similarity search. IEEE/ACM Transactions on Computational Biology and Bioinformatics. 2014;12(4):837–843.

8. Chicco D, Masseroli M. Ontology-based prediction and prioritization of gene functional annotations. IEEE/ACM Transactions on Computational Biology and Bioinformatics. 2015;13(2):248–260.

9. Geistlinger L, Csaba G, Santarelli M, Ramos M, Schiffer L, Turaga N, et al. Toward a gold standard for benchmarking gene set enrichment analysis. Briefings in Bioinformatics. 2021;22(1):545–556.
