# Supplementary material for: Nine quick tips for pathway enrichment analysis
Source: PLoS Comput Biol. 2022 Aug 11;18(8):e1010348. doi: 10.1371/journal.pcbi.1010348 (PMC9371296; doi:10.1371/journal.pcbi.1010348)
Supplement: S2 Text — (PDF) [file pcbi.1010348.s003.pdf]

## S1.3 Usage of different statistical methods in g:Profiler g:GOSt

To further explain the differences in the results that can be achieved with different statistical techniques g:Profiler g:GOSt [1–5] provides three different methods for computing multiple testing correction for $p$-values: g:SCS, Bonferroni correction, or Benjamini-Hochberg false discovery rate [3]. Using these three different statistical tests on the same input gene list can produce different results.

Let us take, for example, the genes of the neuroblastoma prognostic signature proposed by Cangelosi et al. [6–8]. It is a signature of genes that, according to the study authors, can be applied to gene expression to discriminate between survived patients with neuroblastoma and deceased patients with the same child cancer. The signature includes the following non-ranked list of genes: AK4, ALDOC, EGLN1, FAM162A, MTFP1, PDK1, and PGK1. [6].

If we apply g:Profiler g:GOSt (version e104_eg51_p15_3922dba) to this list of genes, with 0.005 threhsold (Tip 5), and use the multiple testing correction method g:SCS, the results contain only nine pathways: seven GO biological processes, one KEGG term ("HIF-1 signaling pathway"), and two WikiPathways. No GO cellular components and GO molecular functions are retrieved. The top associated term is "cellular response to hypoxia" from GO with $p = 5 \times 10^{-6}$.

If we apply g:Profiler g:GOSt to the same neuroblastoma gene list, with the same 0.005 threshold, but with the Bonferroni correction for multiple testing, the results change. In this case, the output contains eleven GO biological processes, one Reactome term ("Gluconeogenesis"), three WikiPathways, and one CORUM term ("HIF1A-OS9-EGLN1 complex").

Again, no GO cellular components and no GO molecular functions are retrieved, and the KEGG "HIF-1 signaling pathway" is present again. The top associated term is again "cellular response to hypoxia" from GO, but this time with a higher $p$-value ($p = 1.7 \times 10^{-6}$).

When we apply g:Profiler g:GOSt to the same gene list, with the same 0.005 threshold, but using the third available test correction method (Benjamini-Hochberg false discovery rate), the results change again. Fourty-five GO biological processes are found this time, together with three KEGG terms: "HIF-1 signaling pathway" is now present with "biosynthesis of amino acids" and "glycolysis / gluconeogenesis". The WikiPathways terms also increased: they are five in this case.

Like the in the Bonferroni correction scenario, no GO cellular components and no GO molecular functions are found. Again, one CORUM element is present ("HIF1A-OS9-EGLN1 complex").

Differently from the other two cases, here for the Benjamini-Hochberg example, the most relevant term result being "cellular response to decreased oxygen levels" of GO with $p = 10^{-6}$. The previously top ranked term "cellular response to hypoxia" is now second in the ranking.

An inexperienced user or a beginner, at this point, might wonder: what statistical method should I use then? The answer, essentially, depends on the study design.

Regarding g:Profiler g:GOSt, an explanation about which correction test to select is given on their website [9].

# References

1. Reimand J, Kull M, Peterson H, Hansen J, Vilo J. g:Profiler—a web-based toolset for functional profiling of gene lists from large-scale experiments. Nucleic Acids Research. 2007;35(suppl_2):W193–W200.

2. Reimand J, Arak T, Vilo J. g:Profiler—a web server for functional interpretation of gene lists (2011 update). Nucleic Acids Research. 2011;39(suppl_2):W307–W315.

3. Reimand J, Arak T, Adler P, Kolberg L, Reisberg S, Peterson H, et al. g:Profiler—a web server for functional interpretation of gene lists (2016 update). Nucleic Acids Research. 2016;44(W1):W83–W89.

4. Kolberg L, Raudvere U, Kuzmin I, Vilo J, Peterson H. gprofiler2–an R package for gene list functional enrichment analysis and namespace conversion toolset g:Profiler. F1000Research. 2020;9.

5. Raudvere U, Kolberg L, Kuzmin I, Arak T, Adler P, Peterson H, et al. g:Profiler: a web server for functional enrichment analysis and conversions of gene lists (2019 update). Nucleic Acids Research. 2019;47(W1):W191–W198.

6. Cangelosi D, Morini M, Zanardi N, Sementa AR, Muselli M, Conte M, et al. Hypoxia predicts poor prognosis in neuroblastoma patients and associates with biological mechanisms involved in telomerase activation and tumor microenvironment reprogramming. Cancers. 2020;12(9):2343.

7. Cangelosi D, Muselli M, Parodi S, Blengio F, Becherini P, Versteeg R, et al. Use of attribute driven incremental discretization and logic learning machine to build a prognostic classifier for neuroblastoma patients. BMC Bioinformatics. 2014;15(5):1–15.

8. Cangelosi D, Pelassa S, Morini M, Conte M, Bosco MC, Eva A, et al. Artificial neural network classifier predicts neuroblastoma patients' outcome. BMC Bioinformatics. 2016;17(12):83–93.

9. g:Profiler. Welcome to g:Profiler; 2022. `https://biit.cs.ut.ee/gprofiler/page/docs#significance_threhshold` URL visited on 2nd February 2022.
