# Supplementary material for: Nine quick tips for pathway enrichment analysis
Source: PLoS Comput Biol. 2022 Aug 11;18(8):e1010348. doi: 10.1371/journal.pcbi.1010348 (PMC9371296; doi:10.1371/journal.pcbi.1010348)
Supplement: S3 Text — (PDF) [file pcbi.1010348.s004.pdf]

S1.4 Pathway data conversion

Pathways differ in the way interactions are modeled, but their data are provided in different formats as well. Standard formats are KEGG Markup Language (KGML) [1], Biological Pathway Exchange (BioPAX) [2] Level 2 and Level 3, System Biology Markup Language (SBML) [3], and the Biological Connection Markup Language (BCML) [4]. KGML [1] provides facilities for computational analysis, drawind and modeling of gene/protein networks, chemical networks, and pathways. BioPAX [2] supports biological pathways representation as well as the many complete basic cellular mechanisms. SBML [3] is a meta language for describing biological models, including signaling pathways, metabolic pathways, gene regulation, and others. BCML [4] is a representation format to describe, annotate and visualize pathways. Pathway information should be provided in a standard format to unify pathway databases. XML is a flexible text format with increasing use for data exchange across different systems. However, XML is very low-level and lacks standard constructs to describe biological phenomena accurately. The BioPAX project is an effort to unify the format and exchange of pathway data. Independent sources such as NCI PID [5], BioCarta [6], Reactome [7], WikiPathways [8], UCSC [9], NIH [10], and others use BioPAX to represent pathway data. As a result, there is currently no accepted standard for constructing pathways. Depending on the database, there may be differences in information sources, experiment interpretation, molecular interactions models, or pathways' boundaries. Hence, the same pathway may have different topologies and representations in other databases. Therefore, all these heterogeneities in the available pathway data limit user analysis because most pathway software tools are designed to use only one pathway database. To analyze pathways coming from not-supported databases, users must manually convert the data in the supported format of the tool, a challenging and error-prone process even for users.

References

1. Klukas C, Schreiber F. Dynamic exploration and editing of KEGG pathway diagrams. Bioinformatics. 2007;23(3):344–350.

2. Demir E, Cary MP, Paley S, Fukuda K, Lemer C, Vastrik I, et al. The BioPAX community standard for pathway data sharing. Nature Biotechnology. 2010;28(9):935–942.

3. Hucka M, Finney A, Sauro HM, Bolouri H, Doyle JC, Kitano H, et al. The systems biology markup language (SBML): a medium for representation and exchange of biochemical network models. Bioinformatics. 2003;19(4):524–531.

4. Beltrame L, Calura E, Popovici RR, Rizzetto L, Guedez DR, Donato M, et al. The Biological Connection Markup Language: a SBGN-compliant format for visualization, filtering and analysis of biological pathways. Bioinformatics. 2011;27(15):2127–2133.

5. Schaefer CF, Anthony K, Krupa S, Buchoff J, Day M, Hannay T, et al. PID: the pathway interaction database. Nucleic Acids Research. 2009;37(suppl_1):D674–D679.

6. Nishimura D. BioCarta. Biotech Software & Internet Report. 2001;2(3):117–120.

7. Joshi-Tope G, Gillespie M, Vastrik I, D'Eustachio P, Schmidt E, de Bono B, et al. Reactome: a knowledgebase of biological pathways. Nucleic Acids Research. 2005;33(suppl_1):D428–D432.

8. Slenter DN, Kutmon M, Hanspers K, Riutta A, Windsor J, Nunes N, et al. WikiPathways: a multifaceted pathway database bridging metabolomics to other omics research. Nucleic Acids Research. 2018;46(D1):D661–D667.

9. Rosenbloom KR, Armstrong J, Barber GP, Casper J, Clawson H, Diekhans M, et al. The UCSC genome browser database: 2015 update. Nucleic Acids Research. 2015;43(D1):D670–D681.

10. O'Keeffe J, Willinsky J, Maggio L. Public access and use of health research: an exploratory study of the National Institutes of Health (NIH) Public Access Policy using interviews and surveys of health personnel. Journal of Medical Internet Research. 2011;13(4):e97.
